# Right atrial function and fibrosis in relation to successful atrial fibrillation ablation

**DOI:** 10.1093/ehjci/jeac152

**Published:** 2022-08-03

**Authors:** Luuk H G A Hopman, Julia E Visch, Pranav Bhagirath, Anja M van der Laan, Mark J Mulder, Orod Razeghi, Michiel J B Kemme, Steven A Niederer, Cornelis P Allaart, Marco J W Götte

**Affiliations:** Department of Cardiology, Amsterdam UMC, Amsterdam, the Netherlands; Department of Cardiology, Amsterdam UMC, Amsterdam, the Netherlands; Department of Cardiology, Amsterdam UMC, Amsterdam, the Netherlands; Department of Cardiology, Amsterdam UMC, Amsterdam, the Netherlands; Department of Cardiology, Amsterdam UMC, Amsterdam, the Netherlands; Division of Imaging Sciences and Biomedical Engineering, King’s College London, London, UK; Department of Cardiology, Amsterdam UMC, Amsterdam, the Netherlands; Division of Imaging Sciences and Biomedical Engineering, King’s College London, London, UK; Department of Cardiology, Amsterdam UMC, Amsterdam, the Netherlands; Department of Cardiology, Amsterdam UMC, Amsterdam, the Netherlands

**Keywords:** atrial remodelling, atrial fibrillation, atrial fibrosis, cardiovascular magnetic resonance (CMR)

## Abstract

**Aims:**

Bi-atrial remodelling in patients with atrial fibrillation (AF) is rarely assessed and data on the presence of right atrial (RA) fibrosis, the relationship between RA and left atrial (LA) fibrosis, and possible association of RA remodelling with AF recurrence after ablation in patients with AF is limited.

**Methods and results:**

A total of 110 patients with AF undergoing initial pulmonary vein isolation (PVI) were included in the present study. All patients were in sinus rhythm during cardiac magnetic resonance (CMR) imaging performed prior to ablation. LA and RA volumes and function (volumetric and feature tracking strain) were derived from cine CMR images. The extent of LA and RA fibrosis was assessed from 3D late gadolinium enhancement images. AF recurrence was followed up for 12 months after PVI using either 12-lead electrocardiograms or Holter monitoring. Arrhythmia recurrence was observed in 39 patients (36%) after the 90-day blanking period, occurring at a median of 181 (interquartile range: 122–286) days. RA remodelling parameters were not significantly different between patients with and without AF recurrence after ablation, whereas LA remodelling parameters were different (volume, emptying fraction, and strain indices). LA fibrosis had a strong correlation with RA fibrosis (*r* = 0.88, *P* < 0.001). Both LA and RA fibrosis were not different between patients with and without AF recurrence.

**Conclusions:**

This study shows that RA remodelling parameters were not predictive of AF recurrence after AF ablation. Bi-atrial fibrotic remodelling is present in patients with AF and moreover, the amount of LA fibrosis had a strong correlation with the amount of RA fibrosis.

## Introduction

Atrial arrhythmogenic remodelling, characterized by changes in atrial structure and function, is central to the development and maintenance of atrial fibrillation (AF).^1^ In addition, atrial fibrosis is increasingly considered an important arrhythmogenic substrate essential for the perpetuation of AF.^2^ The atrial remodelling process is extensively investigated for the left atrium (LA), although recent studies suggest that bi-atrial remodelling is often present in AF.^3–5^ Recently, it has been demonstrated that in congenital heart disease, right atrial (RA) fibrosis is associated with the presence of arrhythmias.^6^ Therefore, it can be postulated that besides LA remodelling, RA remodelling may also contribute to AF perpetuation, and that its presence may affect clinical outcome of pulmonary vein isolation (PVI) ablation.^3^ However, no studies have systematically evaluated the impact of RA remodelling including RA fibrosis on patients with AF and ablative therapy.

Cardiac magnetic resonance (CMR) imaging is well-established for non-invasive assessment of atrial remodelling. Evaluation of atrial function using atrial volumes and atrial feature tracking strain provides insight in atrial remodelling status.^7^ Using 3D late gadolinium enhancement (LGE), the presence and extent of atrial fibrosis can be assessed.^8,9^ A high signal intensity in the atrial wall due to accumulation of contrast agent is considered to be the appearance of fibrotic atrial tissue.^10,11^ Therewith, CMR allows for simultaneous LA and RA remodelling assessment including fibrosis quantification.

In this study, we investigated (i) the presence of RA remodelling and RA fibrosis in patients with AF, (ii) the relation between RA and LA fibrosis, and (iii) predictors of AF recurrence following AF ablation.

## Methods

This retrospective single-centre study was conducted according to the principles outlined in the 1964 Declaration of Helsinki and its later amendments. Follow-up data were collected in a prospectively maintained registry. The local medical ethics committee (VU University Medical Center, Amsterdam, The Netherlands) approved collection and management of data. Written informed consent was obtained from all individual participants included in the study.

### Study population

Patients with paroxysmal or non-paroxysmal AF according to the HRS/EHRA guidelines and scheduled for first radiofrequency (RF) PVI ablation procedure were enrolled in the study between June 2018 and March 2021, partially matching the study cohort presented in previous research.^7^ As part of routine clinical workup, patients underwent a pre-ablation CMR scan to evaluate pulmonary vein anatomy, exclude LA appendage thrombus, and assess atrial fibrosis.

Exclusion criteria to participate in the study were general CMR contraindications, contraindications for a gadolinium-based contrast agent, absence of sinus rhythm during the scan, mechanical heart valves, and a cardiac implantable electronic device.

### CMR acquisition protocol

A detailed CMR protocol has been described previously.^7^ In short, all scans were performed using a 1.5 Tesla clinical magnetic resonance imaging (MRI) system (Siemens AVANTO or SOLA, Erlangen, Germany) using a 32-channel array coil. The CMR protocol included balanced steady-state free precession cine imaging in long axis orientation (two- and four-chamber views). An electrocardiogram (ECG)-gated free-breathing 3D contrast-enhanced MR angiogram (CE-MRA) of the atria and pulmonary veins was obtained during injection of contrast agent (Dotarem®; Guerbet, Roissy, France). High-resolution 3D LGE images were acquired using a navigator-based respiration- and ECG-gated inversion recovery prepared gradient echo-pulse sequence applied approximately 20 min after contrast injection. A regular frequency selective RF pre-pulse was applied to saturate the signal from fat. In-plane resolution was 1.25 × 1.25 mm with slice thickness 2.5 mm (reconstructed to 0.625 × 0.625 × 1.25 mm) for both 3D CE-MRA and 3D LGE images. Depending on the respiratory pattern and the heart rate of the patient, acquisition of the 3D CE-MRA and LGE series took approximately 10–15 min each.

### Image analysis

#### Atrial volume and function assessment

Analysis of cine images was performed using Circle CVI^42^ (Circle Cardiovascular Imaging, Inc, Calgary, Canada). Volumetric data of the LA were derived from the two- and four-chamber cine images using the biplane method. Volumetric data of the RA were derived from the four-chamber cine images. LA minimal volume (LAV_min_) and maximal volume (LAV_max_) as well as RA minimal volume (RAV_min_) and RA maximal volume (RAV_max_) were used to calculate the atrial emptying fractions (LA EF and RA EF, respectively). The indexed atrial volumes were calculated by dividing LAV and RAV by body surface area.

#### Atrial strain analysis

Longitudinal LA and RA feature tracking strain analysis was performed using Circle CVI^42^ Feature Tracking software. Endocardial and epicardial borders of the LA and RA were traced in the end-diastolic phase in the two- and four-chamber cine images (see Supplementary data online, *Figure S1*). An automated tracking algorithm was used and manual adjustments were applied if the atrial wall was not followed properly. Longitudinal strain measurements were subdivided into reservoir strain, conduit strain, and contractile strain.

#### Atrial fibrosis quantification

Quantification of LA and RA fibrosis was performed using open-source Cardiac Electro-Mechanics Research Group (CEMRG) image post-processing software (King’s College London, London, UK).^12^ The 3D LGE images underwent stringent quality control (i.e. artefacts, proper myocardial nulling) by two experienced readers prior to post-processing and images were excluded from analysis if quality was deemed insufficient. Using CEMRG, the LA blood pool (including PV extensions) and RA blood pool (including inferior vena cava, superior vena cava, and coronary sinus) were segmented semi-automatically in the 3D CE-MRA images on axial slices using a thresholding tool. The interpolated contours were adjusted manually in each axial plane if necessary. A two-voxel (1.25 mm) surface dilation was used to define the epicardial border. Subsequently, the 3D CE-MRA was co-registered with the 3D LGE images and a manual correction for misregistration was performed if necessary. Thereafter, 3D reconstructions of the LA and RA were generated. The LA appendage and the pulmonary veins were manually excluded from the 3D LA segmentation. The mitral valve annulus was used as landmark to separate the LA from the LV cavity. From the 3D RA model, the inferior vena cava, superior vena cava, and coronary sinus were manually excluded at their ostia and the tricuspid valve annulus was used to separate the RA from the RV cavity.

On the 3D LGE images, signal intensity was normalized to the mean blood pool intensity according to the image intensity ratio (IIR) method and a threshold of 1.2 was used to indicate fibrosis (see Supplementary data online, *Figure S2*).^8,13^

### Ablation procedure and arrhythmia follow-up

Ablation procedures were performed according to standard protocol under conscious sedation, deep sedation, or general anaesthesia. RF lesions were created in a wide-area circumferential ablation pattern to achieve isolation of the pulmonary veins, and PVI durability was assessed after a waiting period of at least 30 min. No additional LA ablation lines were placed (i.e. roof line, posterior wall box lesion). In some patients, a cavotricuspid isthmus (CTI) ablation was performed as part of the same PVI ablation procedure.

Patients were followed up in the outpatient clinic for 12 months after PVI. A minimum of four ECGs were recorded at 1, 3, 6, and 12 months follow-up in all patients, and additional ECG recordings or 24–48 h Holter monitoring were obtained in patients who experienced symptoms suggestive of tachyarrhythmia recurrence. Antiarrhythmic drugs were typically discontinued after 3 months post-ablation. Any documented episode of AF, atrial flutter, or atrial tachycardia lasting >30 s was considered early recurrence during the blanking period of 90 days post-ablation, and AF recurrence thereafter.

### Statistical analysis

Results are presented as mean ± standard deviation for normally distributed data and median including interquartile range (IQR) for data with a non-normal distribution. Normality of continuous data was assessed by inspection of histograms and Q-Q plots. Where appropriate, continuous variables were compared using either an independent samples *t*-test or Mann–Whitney *U* test. Pearson’s correlation was used to quantify associations between continuous variables. Kaplan–Meier analyses were performed to assess freedom of recurrence. Logistic regression was used to identify parameters predicting the recurrence of AF. After performing univariable analysis, multivariable regression was performed using backward elimination. Predictive parameters with *P*-values <0.1 were considered for the multivariable analysis. Inter- and intraobserver variabilities were assessed in 10 randomly selected patients to test for reproducibility of LA and RA fibrosis quantification in which the entire segmentation process was redone, including exclusion of the veins and valve annulus (L.H.G.A.H. and J.E.V.). Agreement between measurements was assessed by intraclass correlation coefficients (ICCs) and visually by Bland–Altman analysis. ICCs for absolute agreement of single measurements were estimated using a two-way random effect model. Data were considered significant if *P*-value <0.05. Statistical analysis was performed using SPSS Statistics v26 (IBM Corporation, Armonk, NY, USA).

## Results

A total of 110 patients with AF were included in the study. Of those, four patients (4%) had no 3D LGE imaging and eight patients (7%) had insufficient LGE image quality for atrial fibrosis assessment due to inadequate myocardial nulling, motion/ghosting artefacts, or inflow artefacts. Baseline characteristics of the study participants are presented in *Table 1*. Two-third of the patient population was male and the mean age was 60 ± 9 years. Paroxysmal AF was present in 79 patients (72%) and the median time between AF diagnosis and the CMR scan was 33 (14–76) months.

**Table 1:** Baseline characteristics of the study population

Demographics	
Age, years	60 ± 9
Male gender	72 (66%)
Weight (kg)	85 ± 14
Height (cm)	180 ± 10
BMI (kg/m^2^)	26.2 ± 3.7
BSA (Mosteller)	2.1 ± 0.2
CHA_2_DS_2_-VASc score ≥2	39 (35%)
Hypertension	37 (34%)
Diabetes mellitus	5 (5%)
Congestive heart disease	4 (3.6%)
Coronary artery disease	8 (7.3%)
*Medications*	
ACE inhibitor or ARB	33 (30%)
Spironolactone	2 (2%)
Amiodarone	11 (10%)
Anticoagulation	91 (83%)
*AF history*	
Paroxysmal AF	79 (72%)
Persistent AF	31 (28%)
Time between AF diagnosis and CMR (months)	33 (14–76)
PVI + CTI ablation	21 (19%)

Values are expressed as number (percentage), mean ± SD or median (25th–75th percentile).

ACE, angiotensin-converting-enzyme; ARB, angiotensin-receptor blocker; AF, atrial fibrillation; BMI, body mass index; BSA, body surface area calculated by the Mosteller method {[height (cm) × weight (kg)/3600]^½^}.

### Atrial remodelling in patients with AF

**Figure 1:**
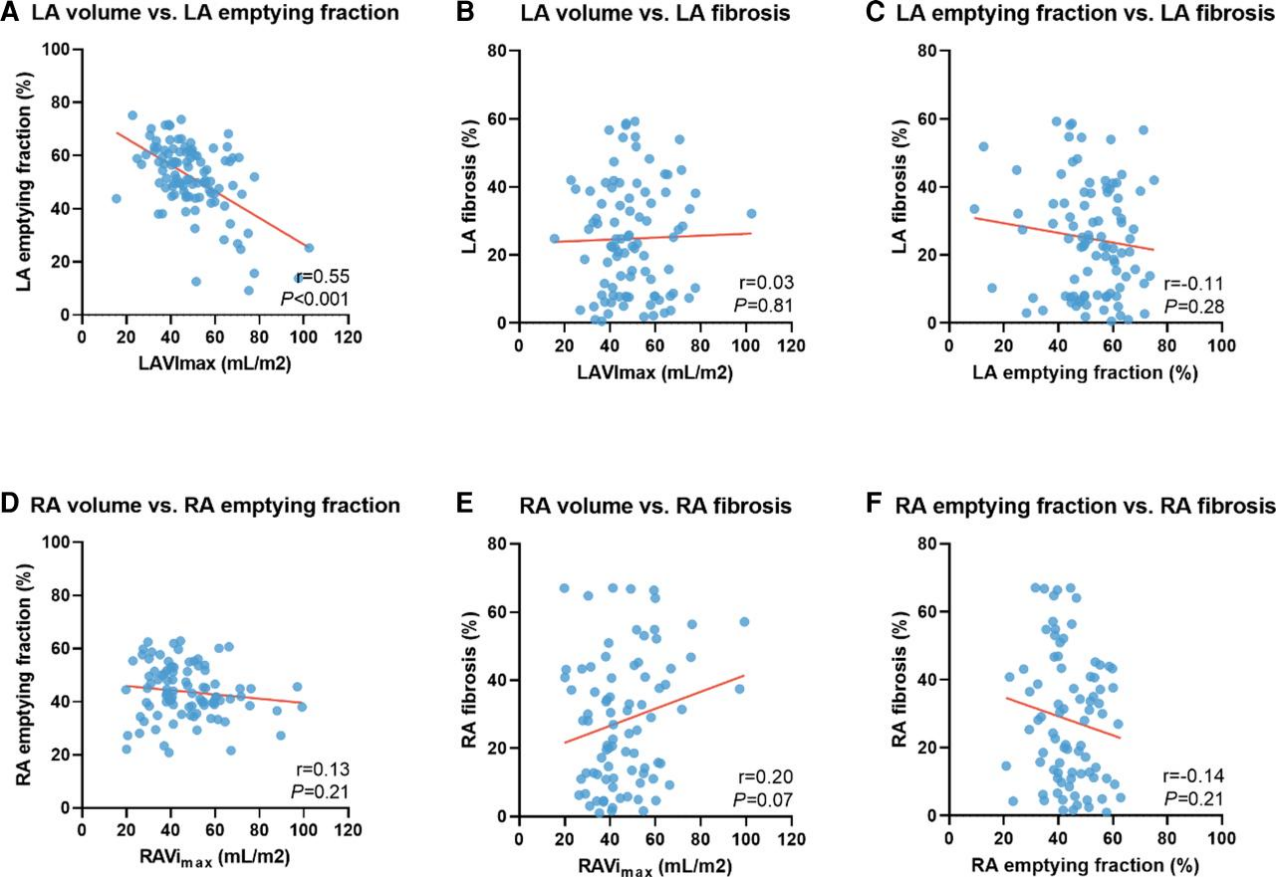
Correlation between atrial remodelling indices. Scatterplots comparing (*A*) maximal left atrial volume index and left atrial emptying fraction, (*B*) maximal left atrial volume index and left atrial fibrosis, (*C*) left atrial emptying fraction and left atrial fibrosis, (*D*) maximal right atrial volume index and right atrial emptying fraction, (*E*) maximal right atrial volume index and right atrial fibrosis, and (*F*) right atrial emptying fraction and right atrial fibrosis.

The median time between CMR scan and AF ablation procedure was 32 days (IQR: 14–77 days). LAVi_max_ was 49.48 ± 14.56 mL/m^2^ and LA EF was 51.75 ± 13.23%. Mean LA fibrosis was 24.36 ± 16.14% and there was no correlation between LA fibrosis and LAVi_max_ (*r* = 0.03, *P* = 0.81) or LA fibrosis and LA EF (*r* = 0.11, *P* = 0.28; *Figure 1*). A weak but significant relation was found between LA fibrosis and LA reservoir strain (*r* = 0.29, *P* < 0.01), and LA conduit strain (*r* = 0.31, *P* < 0.01), but not with LA contractile strain (*r* = 0.10, *P* = 0.33).

RAVi_max_ was 47.01 ± 16.35 mL/m^2^ and RA EF was 43.77 ± 9.92%. Mean RA fibrosis was 28.60 ± 19.48% and also for the RA, the amount of fibrosis was not correlated with either RAVi_max_ or RA EF (*r* = 0.20, *P* = 0.07 and *r* = −0.14, *P* = 0.21, respectively; *Figure 1*) but a significant relation was found between RA fibrosis and RA reservoir strain (*r* = 0.28, *P* = 0.02).

The duration of AF was not related with the amount of LA and RA fibrosis (LA: *P* = 0.96, RA: *P* = 0.49). Patients undergoing a CTI ablation as part of the same PVI ablation procedure did not have a higher RA fibrotic burden when compared with patients undergoing PVI alone (29.84 ± 19.06 vs. 28.28 ± 19.70%, *P* = 0.76). Moreover, also RA volume, RA EF, and RA strain indices were not different between these two groups.

### LA vs. RA remodelling in patients with AF

**Figure 2:**
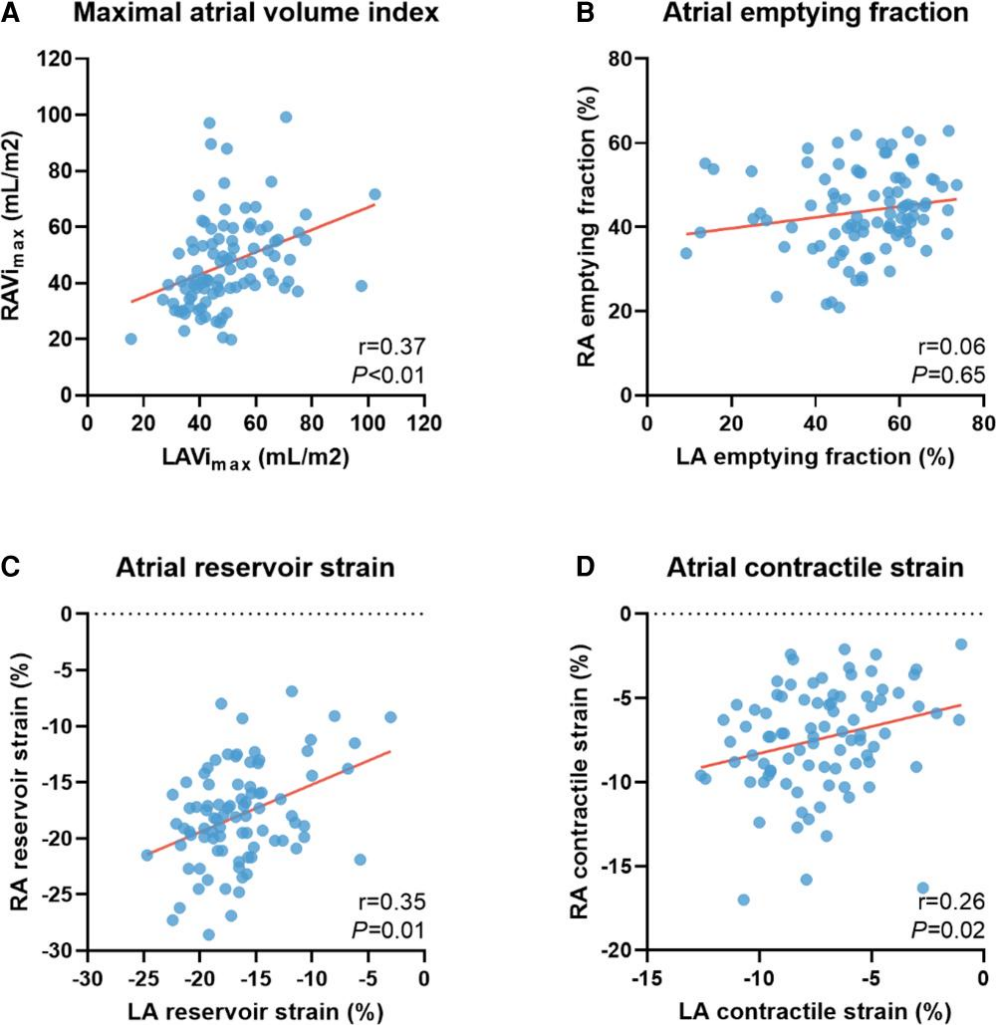
Correlation between left atrial and right atrial remodelling indices. Scatterplot comparing (*A*) maximal left atrial volume index and maximal right atrial volume index, (*B*) left atrial emptying fraction and right atrial emptying fraction, (*C*) left atrial reservoir strain and right atrial reservoir strain, and (*D*) left atrial contractile strain and right atrial contractile strain.

**Figure 3:**
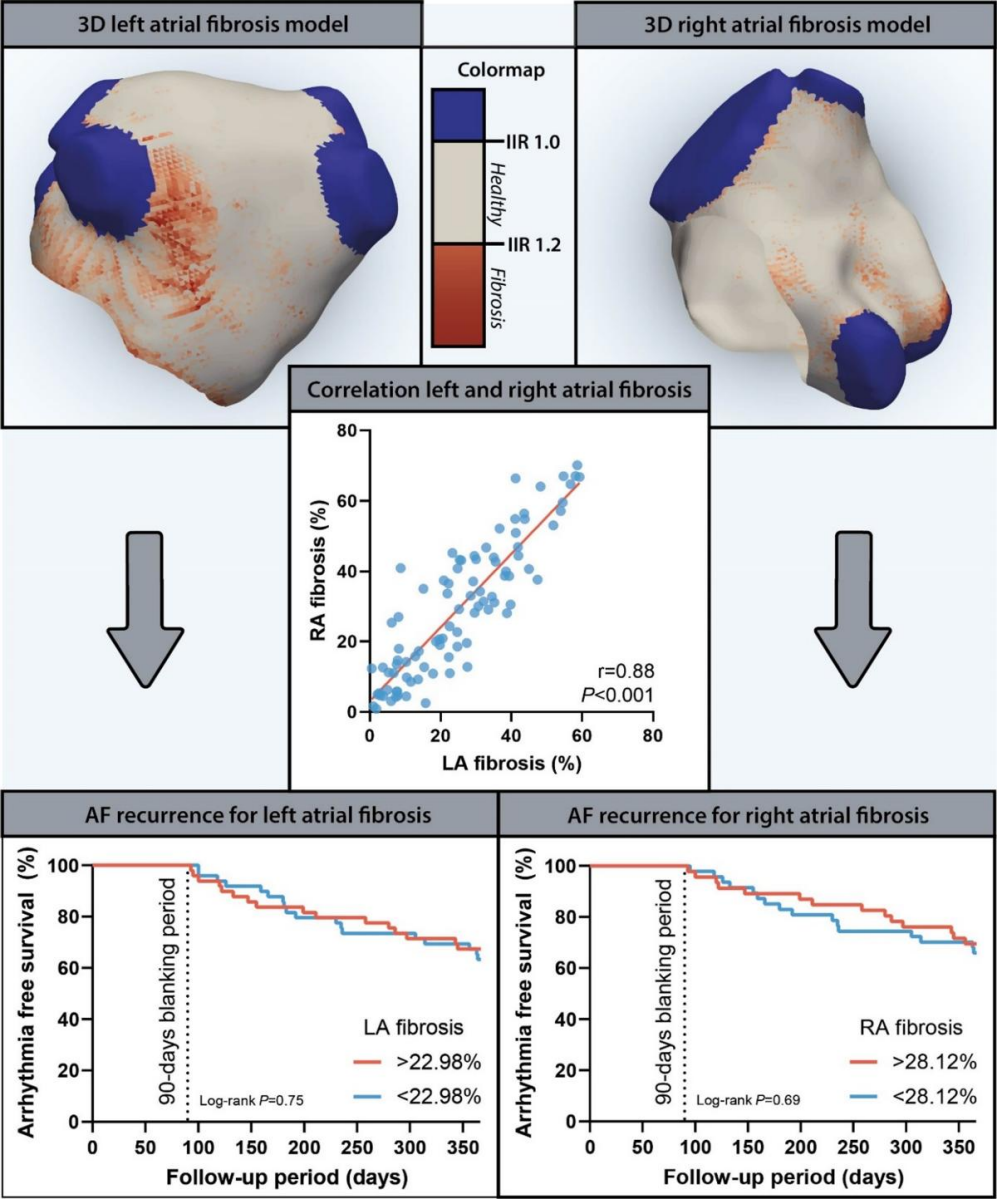
Central figure: Left atrial fibrosis and right atrial fibrosis in relation to each other and AF recurrence after AF ablation. Demonstration of a 3D LA and RA fibrosis model. A scatterplot demonstrating the correlation between LA fibrosis and RA fibrosis is depicted in the middle. Kaplan–Meier curves showing the arrhythmia free survival after one year follow-up for LA and RA fibrosis (divided by the median fibrosis value) are depicted below.

LA and RA characteristics are listed in Supplementary data online, *Table S1*. LAVi_max_ was correlated with RAVi_max_ (*r* = 0.36, *P* < 0.001), whereas LA EF was not correlated with RA EF (*r* = 0.18, *P* = 0.09; *Figure 2*). There was a significant correlation between LA and RA reservoir strain (*r* = 0.39, *P* < 0.001), conduit strain (*r* = 0.45, *P* < 0.001), and contractile strain (*r* = 0.26, *P* = 0.02). LA fibrosis had a strong correlation with RA fibrosis, with a Pearson correlation coefficient of 0.88 (*P* < 0.001; *Figure 3*).

### LA and RA remodelling in relation to AF recurrence after ablation

Arrhythmia recurrence after the 90-day blanking period was observed in 39 of the 110 patients (36%), occurring over a median of 181 (IQR: 122–286) days.

In *Table 2*, characteristics were compared between the patients with and without AF recurrence after ablation. There was no difference in age, gender, and AF duration between both groups. A trend towards significance for more recurrence in patients with non-paroxysmal AF when compared with paroxysmal AF was observed (48 vs. 30%, *P* = 0.08, respectively). LVEF was not significantly different between the two groups (AF recurrence: 56.73 ± 9.11 vs. no AF recurrence: 59.43 ± 6.77, *P* = 0.12).

**Table 2:** Characteristics of patients with AF recurrence and without recurrence after catheter ablation

	*Recurrence of AF* *(n = 39)*	*No recurrence of AF* *(n = 71)*	*P-value*
Age (years)	59 ± 9	60 ± 9	0.50
Male gender, *n* (%)	24 (62%)	48 (68%)	0.52
BMI (kg/m^2^)	26.7 ± 4.1	25.9 ± 3.5	0.30
Hypertension, *n* (%)	17 (44%)	20 (28%)	0.10
Non-paroxysmal AF, *n* (%)	15 (38%)	16 (22%)	0.08
AF duration (months)	35 [14–101]	32 [14–71]	0.55
PVI + CTI ablation	6 (15%)	15 (21%)	0.44
*Left atrial volume*			
Volume: min (mL)	61.73 ± 32.65	45.26 ± 22.86	**<0**.**01**
Volume: max (mL)	111.20 ± 32.21	96.65 ± 29.74	**0**.**02**
Stoke volume (mL)	49.47 ± 17.78	51.39 ± 15.40	0.57
Emptying fraction (%)	46.25 ± 14.55	54.54 ± 11.60	**<0**.**01**
Volume index: min (mL/m^2^)	30.55 ± 16.01	22.08 ± 11.48	**<0**.**01**
Volume index: max (mL/m^2^)	54.67 ± 14.50	46.92 ± 13.94	**<0**.**01**
*Left atrial strain*			
Reservoir strain (%)	−14.67 ± 4.49	−16.97 ± 3.47	**<0**.**01**
Conduit strain (%)	−8.48 ± 2.74	−9.02 ± 2.95	0.38
Contractile strain (%)	−6.49 ± 2.60	−7.95 ± 2.21	**<0**.**01**
Peak positive strain rate (%)	0.61 ± 0.18	0.76 ± 0.22	**0**.**001**
Peak early negative strain rate (%)	−0.76 ± 0.29	−0.85 ± 0.27	0.12
Peak late positive strain rate (%)	−0.68 ± 0.27	−0.91 ± 0.29	**<0**.**001**
Left atrial fibrosis (%)	23.07 ± 16.55	25.22 ± 15.95	0.40
Left atrial sphericity (%)	79.86 ± 3.22	79.35 ± 2.87	0.53
*Right atrial volume*			
Volume: min (mL)	58.37 ± 24.56	51.68 ± 21.88	0.18
Volume: max (mL)	101.21 ± 35.46	93.53 ± 32.43	0.29
Stoke volume (mL)	42.84 ± 17.03	41.84 ± 15.45	0.77
Emptying fraction (%)	42.42 ± 10.24	44.53 ± 9.74	0.32
Volume index: min (mL/m^2^)	28.59 ± 11.78	25.14 ± 10.89	0.16
Volume index: max (mL/m^2^)	49.65 ± 16.90	45.52 ± 15.98	0.24
*Right atrial strain*			
Reservoir strain (%)	−17.45 ± 4.13	−18.20 ± 4.65	0.45
Conduit strain (%)	−10.03 ± 3.54	−11.02 ± 4.13	0.23
Contractile strain (%)	−7.42 ± 3.16	−7.18 ± 3.69	0.76
Peak positive strain rate (%)	0.89 ± 0.33	0.94 ± 0.24	0.38
Peak early negative strain rate (%)	−0.87 ± 0.32	−0.95 ± 0.29	0.23
Peak late positive strain rate (%)	−0.81 ± 0.39	−0.82 ± 0.33	0.93
Right atrial fibrosis (%)	27.79 ± 20.61	28.98 ± 19.08	0.79
Right atrial sphericity (%)	78.18 ± 2.91	78.30 ± 2.86	0.86

Values are expressed as mean ± SD.

AF, atrial fibrillation; BMI, body mass index; CMR, cardiovascular magnetic resonance imaging; LA, left atrial; RA, right atrial.

**Figure 4:**
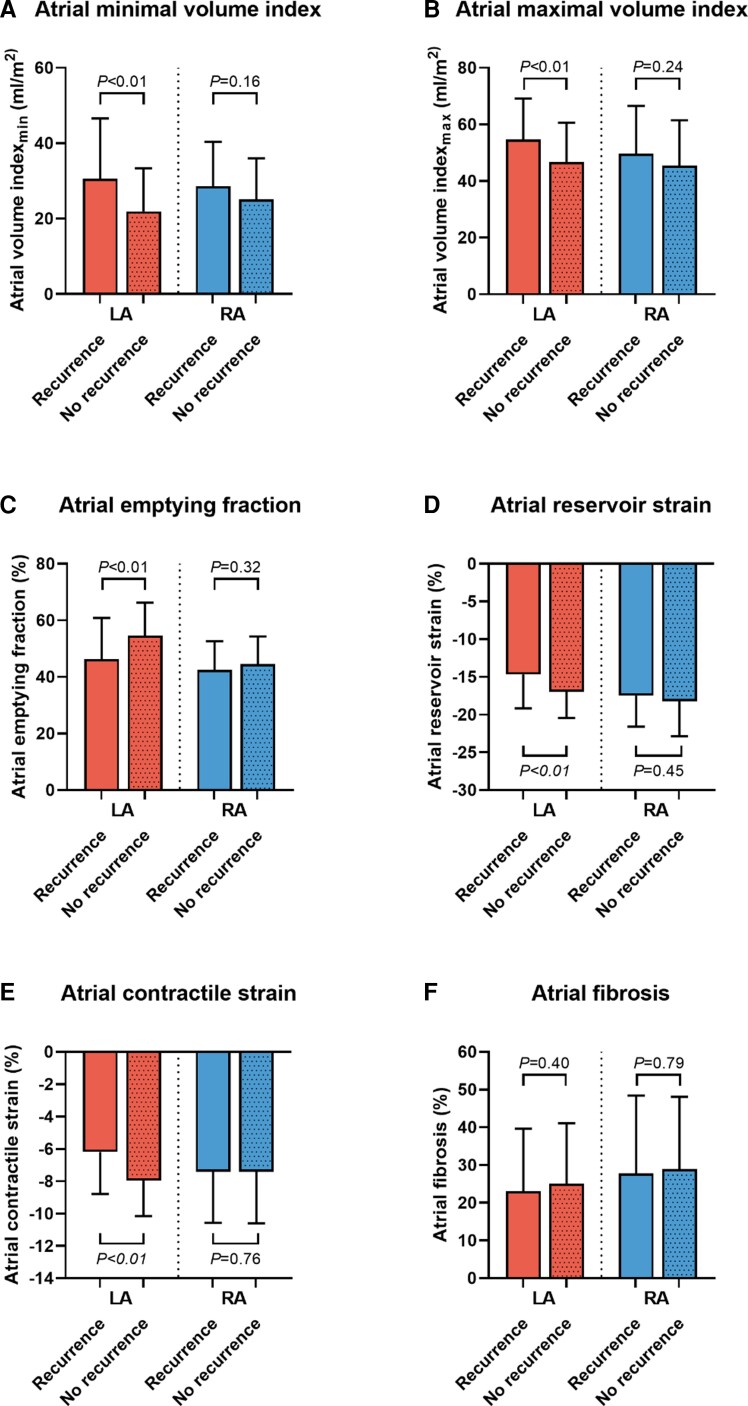
Left and right atrial remodelling indices in relation to AF recurrence. Bar graphs demonstrating the difference in AF recurrence for LA and RA volumes (*A* and *B*), emptying fraction (*C*), reservoir strain (*D*), contractile strain (*E*), and fibrosis (*F*).

In contrast to LA parameters, RA remodelling parameters were not significantly different between patients with and without AF recurrence after ablation (*Table 2*). LAVi_max_ was significantly higher in patients with AF recurrence (54.67 ± 14.50 vs. 46.92 ± 13.94 mL/m^2^, *P* < 0.01) and LA EF was significantly lower in patients with AF recurrence (46.25 ± 14.55 vs. 54.54 ± 11.60%, *P* < 0.01) when compared with patients without recurrence (*Figure 4*). LA reservoir strain and LA contractile strain were significantly impaired in patients with AF recurrence compared with patients without AF recurrence (LA reservoir strain; −14.67 ± 4.49 vs. −16.97 ± 3.47%, *P* < 0.01 and LA contractile strain; −6.49 ± 2.60 vs. −7.95 ± 2.21%, *P* < 0.01, respectively; *Figure 4*). LA fibrosis was not different between patients with and without recurrence of AF (23.07 ± 16.55 vs. 25.22 ± 15.95, *P* = 0.40, respectively; *Figure 4*). Also, RA fibrosis was not different between the two groups (27.79 ± 20.61 vs. 28.98 ± 19.08%, *P* = 0.79).

Based on the median LA fibrosis (22.98%) and RA fibrosis (28.12%) values, survival curves were created and displayed in *Figure 3*. Kaplan–Meier analysis demonstrated no significant difference in recurrence of AF between high fibrosis (above median value) and low fibrosis (below median value) for both LA and RA (LA: Log-rank *P* = 0.75, RA: Log-rank *P* = 0.69). Lastly, Supplementary data online, *Table S2* presents the univariable and multivariable analyses for the association of clinical and imaging parameters with AF recurrence after ablation. Among the parameters associated with AF recurrence, LA contractile strain was independently associated with AF recurrence during follow-up (*P* < 0.001).

### Reproducibility

A total of 10 randomly selected patients underwent repeated review to assess intra- and interobserver reliability for atrial fibrosis quantification. The ICC for inter-reader variability of LA fibrosis 0.90 (95% confidence interval: 0.76–0.96). The ICC for intrareader variability of LA fibrosis was 0.96 (95% confidence interval: 0.87–0.99). For RA fibrosis, the ICC for inter-reader variability was 0.97 (95% confidence interval: 0.87–0.99) and the ICC for intrareader variability was 0.96 (95% confidence interval: 0.85–0.99).

## Discussion

This study showed that in patients with AF undergoing PVI ablation, atrial fibrosis assessed using LGE-CMR was found in equal amounts for the LA and RA. Moreover, the degree of LA fibrosis had a very strong correlation with the degree of RA fibrosis albeit the presence of fibrosis was not predictive for AF recurrence after AF ablation.

RA remodelling parameters were not significantly different between patients with and without AF recurrence after 1 year follow-up. Only LA remodelling parameters (volume and function) were significantly different between the two groups, and LA contractile strain was an independent predictor of AF recurrence. Therefore, the present study suggests that predominantly LA remodelling is associated with procedural success, although large-scale studies are needed to confirm our results.

### Right atrial remodelling in AF

Literature on the relationship between RA remodelling and AF is limited and the clinical consequences of RA remodelling in development of AF remain largely unresolved. In a Multi-Ethnic Study of Atherosclerosis substudy, higher RA volume indices were associated with incident AF but no association of RA function (global longitudinal strain or RA EF) with AF was found after adjustment for risk factors and subclinical cardiovascular disease.^14^ Moreover, the presence and role of RA remodelling in AF recurrence after ablation remains debatable. Akutsu and colleagues^3^ demonstrated that both LA and RA remodellings are equally associated with AF recurrence after ablative therapy. This finding was in contrast to the results of a study by Shin *et al*.,^15^ in which LAVi was the only independent predictor for AF recurrence, whereas RA remodelling parameters were not. Likewise, in the present study, only LA remodelling parameters were different between patients with AF experiencing AF recurrence after ablation and patients without AF recurrence. Accordingly, in our patient cohort, LA and not RA remodelling status appears to be associated with AF patients at higher risk for recurrence after PVI. Nevertheless, various LA remodelling indices were correlated with RA remodelling indices. A significant correlation between LA and RA volumes, and LA and RA strain indices was found in the present study. This indicates that there is an interdependency between the two atrial compartments, although the relation between these symmetrical atrial alterations in the context of the AF disease process and possible reverse bi-atrial remodelling following PVI needs further investigation.

### Atrial fibrosis in AF

Atrial fibrosis assessed using LGE-CMR was present in both the LA and RA. Interestingly, the amount of LA and RA fibrosis was almost identical in both compartments and correlation between the two was found to be very strong. These findings are in line with observations by O’Neill and colleagues.^6^ In their study, a linear relationship was observed between LA fibrosis and RA fibrosis burden in a subgroup of paroxysmal patients with AF. This relationship suggests that AF might promote structural remodelling in both atria leading to a bi-atrial pathology. Histological assessment contributes to this suggestion as demonstrated by Kainuma and colleagues.^16^ In patients undergoing a surgical AF ablation approach (maze procedure), quantitative measurements of fibrosis areas revealed a comparable amount of atrial fibrosis in the LA and RA. Therewith, progressive electrophysiological remodelling including development of fibrosis does not appear to be an isolated LA phenomenon, but rather a process encompassing both atria. Hence, hypothesizing that not only LA fibrosis but also RA fibrosis potentially serves as an arrhythmogenic substrate for AF maintenance.

### Atrial remodelling and clinical implications

AF might be considered an atrial cardiomyopathy instigating the development of bi-atrial fibrosis. The extent of atrial fibrotic remodelling is related to the risk of unsuccessful treatment in patients undergoing AF ablation as demonstrated in the DECAAF trial.^9^ Recent studies, however, have difficulties reproducing this relationship^17–19^ and research on MRI-guided ablation of LA-LGE areas in addition to PVI failed to show improved outcome in comparison with a PVI-only approach.^20^ Also in the present study, no association between the amount of LA or RA fibrosis and the recurrence risk after AF ablation was found. Therefore, one could argue that atrial fibrosis may be a manifestation of the atrial cardiomyopathy, but not the hallmark driver of AF. Supportive for this hypothesis is the observation that no relationship was found between the amount of fibrosis and AF duration, or the amount of fibrosis and clinical types of AF. Alternatively, the diagnostic accuracy of LGE-CMR to detect atrial fibrosis might be suboptimal. A recent histological study in patients with AF by Maesen *et al*.^21^ showed that endomysial fibrosis (within muscle bundles) correlated well with AF complexity, while this relation was not found for total fibrosis, which also incorporates fibrosis between muscle bundles. This particular distinction in fibrosis types cannot be made by LGE-CMR and hence it might also explain the mismatch between LGE-CMR measured fibrosis and invasive high-density voltage and activation mapping as a surrogate marker of atrial cardiomyopathy.^17,22^

Functional remodelling parameters such as LA volumetric measures and LA phasic strain, however, have shown to be strong predictors of AF recurrence.^23^ Reduced LA deformation accompanied with severe atrial dilatation seems to be an unfavourable condition for successful AF ablative therapy. In our study cohort, LA volumetric and functional remodelling parameters were different between patients experiencing AF recurrence and patients who remained free of AF after ablation. Of those parameters, LA contractile strain tends to be the most valuable marker for prediction of AF recurrence as demonstrated by multivariable analysis.

## Limitations

Firstly, atrial fibrosis quantification using LGE-CMR has several inherent limitations. Differences in CMR acquisition such as contrast dosage, interval between contrast administration and image acquisition, scanner field strength, voxel size, image acquisition parameters, inversion time choice, and patient-specific clearance rate might impact the detection of atrial fibrosis.^24^ These factors could explain that the mean fibrotic burden measured in our cohort is relatively high, although not exceptional compared with values found in literature.^9,25,26^ Moreover, quantification methods and thresholding values could also impact measured fibrotic burden.^10,17^ In the present study, the IIR method with a threshold value of 1.2 was chosen to assess atrial fibrosis. However, there is currently no complete agreement on IIR thresholds for LA-LGE since direct histological validation on specific IIR cut-off values is lacking.^10^ Additionally, we used a fixed wall segmentation thickness of two pixels as per default setting for both LA and RA. However, the thin atrial wall has an alternating thickness. Therefore, any partial volume effects could not be excluded, although we aimed to mitigate artefacts by performing the segmentation on the 3D CE-MRA which provides higher contrast for identifying the endocardium.^27^ Furthermore, LGE-CMR-based RA fibrosis detection is rarely performed and it is unknown if similar segmentation and thresholding techniques can be used in both atria.

Secondly, RA volumes and strain were calculated from the four-chamber cine images only, while LA volumes and strain were calculated from two- and four-chamber cine images using the biplane method. As a consequence, RA remodelling assessment might be less accurate which may have impacted the results.

Lastly, arrhythmia recurrence was based on documentation of 12-lead ECGs or Holter monitoring at routine intervals or when triggered by patients’ symptoms. No continuous monitoring of AF was executed in this study and asymptomatic AF recurrence may have been missed which may have caused underestimation of AF prevalence.

## Conclusion

Bi-atrial fibrotic remodelling is present in patients with AF and the amount of LA fibrosis had a strong correlation with the amount of RA fibrosis. The presence of bi-atrial fibrotic tissue therefore may be an expression of disease state, although no relation with AF recurrence after AF ablation was found. Larger studies investigating the bi-atrial interplay in fibrosis development and the potential role in arrhythmogenesis are desired.

## Supplementary data

Supplementary data are available at *European Heart Journal – Cardiovascular Imaging* online.

## Supplementary Material

jeac152_Supplementary_Data

## Data Availability

The data underlying this article will be shared on reasonable request to the corresponding author.
