# Supplementary material for: Right atrial function and fibrosis in relation to successful atrial fibrillation ablation
Source: Eur Heart J Cardiovasc Imaging. 2022 Aug 3;24(3):336–45. doi: 10.1093/ehjci/jeac152 (PMC9936834; doi:10.1093/ehjci/jeac152)
Supplement: jeac152_Supplementary_Data [file jeac152_supplementary_data.docx]

**SUPPLEMENTARY MATERIAL**

**Right Atrial Function and Fibrosis in Relation to Successful Atrial Fibrillation Ablation**

Luuk H.G.A. Hopman, Msc; Julia Visch, Bsc; Pranav Bhagirath, MD, PhD; Anja M. van der Laan, MD, PhD; Mark J. Mulder, MD; Orod Razeghi, PhD; Michiel J.B. Kemme, MD, PhD; Steven Niederer, PhD; Cornelis P. Allaart, MD, PhD; Marco J.W. Götte, MD, PhD

**Supplementary tables:**

**Table S1.** Correlation between LA and RA characteristics in AF patients.

**Table S2.** Univariable and multivariable backwards stepwise logistic regression analysis for parameters associated with AF recurrence

**Supplementary figures:**

**Figure S1.** Left and right atrial strain assessment**.**

**Figure S2.** Left and right atrial fibrosis quantification.

| **Table S1:** Correlation between LA and RA characteristics in AF patients | | | | |
| --- | --- | --- | --- | --- |
|  | **Left atrium** | **Right atrium** | **Pearson correlation** | **P-value** |
| Atrial volume |  |  |  |  |
| Volume - min (ml) | 50.77 ± 27.66 | 54.10 ± 22.98 | 0.20 | 0.05 |
| Volume - max (ml) | 101.43 ± 31.26 | 96.31 ± 33.57 | 0.39 | **<0.001** |
| Emptying fraction (%) | 51.75 ± 13.23 | 43.77 ± 9.92 | 0.18 | 0.09 |
| Volume index - min (ml/m^2^) | 24.92 ± 13.76 | 26.39 ± 11.28 | 0.21 | **0.04** |
| Volume index - max (ml/m^2^) | 49.48 ± 14.56 | 47.01 ± 16.35 | 0.36 | **<0.001** |
| Atrial strain |  |  |  |  |
| Reservoir strain (%) | -16.18 ± 3.98 | -17.92 ± 4.45 | 0.39 | **<0.001** |
| Conduit strain (%) | -8.84 ± 2.87 | -10.65 ± 3.68 | 0.45 | **<0.001** |
| Contractile strain (%) | -7.35 ± 2.48 | -7.27 ± 3.48 | 0.26 | **0.02** |
| Peak positive strain rate (%) | 0.71 ± 0.21 | 0.92 ± 0.27 | 0.24 | **0.03** |
| Peak early negative strain rate (%) | -0.82 ± 0.28 | -0.92 ± 0.30 | 0.40 | **<0.001** |
| Peak late negative strain rate (%) | -0.83 ± 0.30 | -0.82 ± 0.35 | 0.33 | **<0.01** |
| Atrial fibrosis (%) | 24.36 ± 16.14 | 28.60 ± 19.48 | 0.88 | **<0.001** |
| Atrial sphericity (%) | 79.53 ± 3.00 | 78.26 ± 2.86 | -0.14 | 0.19 |
| Values are expressed as mean ± SD. AF, atrial fibrillation; CMR, cardiovascular magnetic resonance imaging; LA, left atrial; RA, right atrial. | | | | |

| Table S2. Univariable and multivariable backwards stepwise logistic regression analysis for parameters associated with AF recurrence | | | | |
| --- | --- | --- | --- | --- |
| Dependent variable AF recurrence during follow-up | **Univariable** | | **Multivariable** | |
|  | OR (95% CI) | *P*-value | OR (95% CI) | *P-*value |
| Age | -0.00 (-0.01 – 0.01) | 0.50 |  |  |
| Male gender | -0.06 (-0.25 -0.13) | 0.53 |  |  |
| BMI | 0.01 (-0.01 -0.04) | 0.30 |  |  |
| Non-paroxysmal AF | 0.18 (-0.02 -0.38) | 0.08 | - | - |
| Hypertension | 0.16 (-0.03 – 0.35) | 0.103 |  |  |
| Congestive heart disease | 0.29 (-0.06 – 0.64) | 0.099 | - | - |
| Coronary artery disease | -0.37 (-0.85 – 0.11) | 0.13 |  |  |
| PVI + CTI ablation | -0.09 (-0.32 – 0.14) | 0.45 |  |  |
| LA volume index - max | -0.01 (-0.02 – 0.001) | 0.08 | - | - |
| RA volume index - max | 0.00 (-0.00 – 0.10) | 0.24 |  |  |
| LA EF | -0.01 (-0.02 -0.00) | <0.01 | - | - |
| RA EF | -0.01 (-0.02 – 0.01) | 0.32 |  |  |
| LA contractile strain | 0.07 (0.03 – 0.10) | <0.001 | 0.07 (0.03 – 0.10) | <0.001 |
| RA contractile strain | 0.00 (-0.03 – 0.03) | 0.99 |  |  |
| LA fibrosis | -0.00 (-0.01 – 0.00) | 0.57 |  |  |
| RA fibrosis | -0.00 (-0.01 – 0.00) | 0.79 |  |  |
| Predictors were considered for the multivariable analysis when p<0.1. AF, atrial fibrillation; BMI, body mass index; EF, ejection fraction; LA, left atrial; RA, right atrial. | | | | |

**
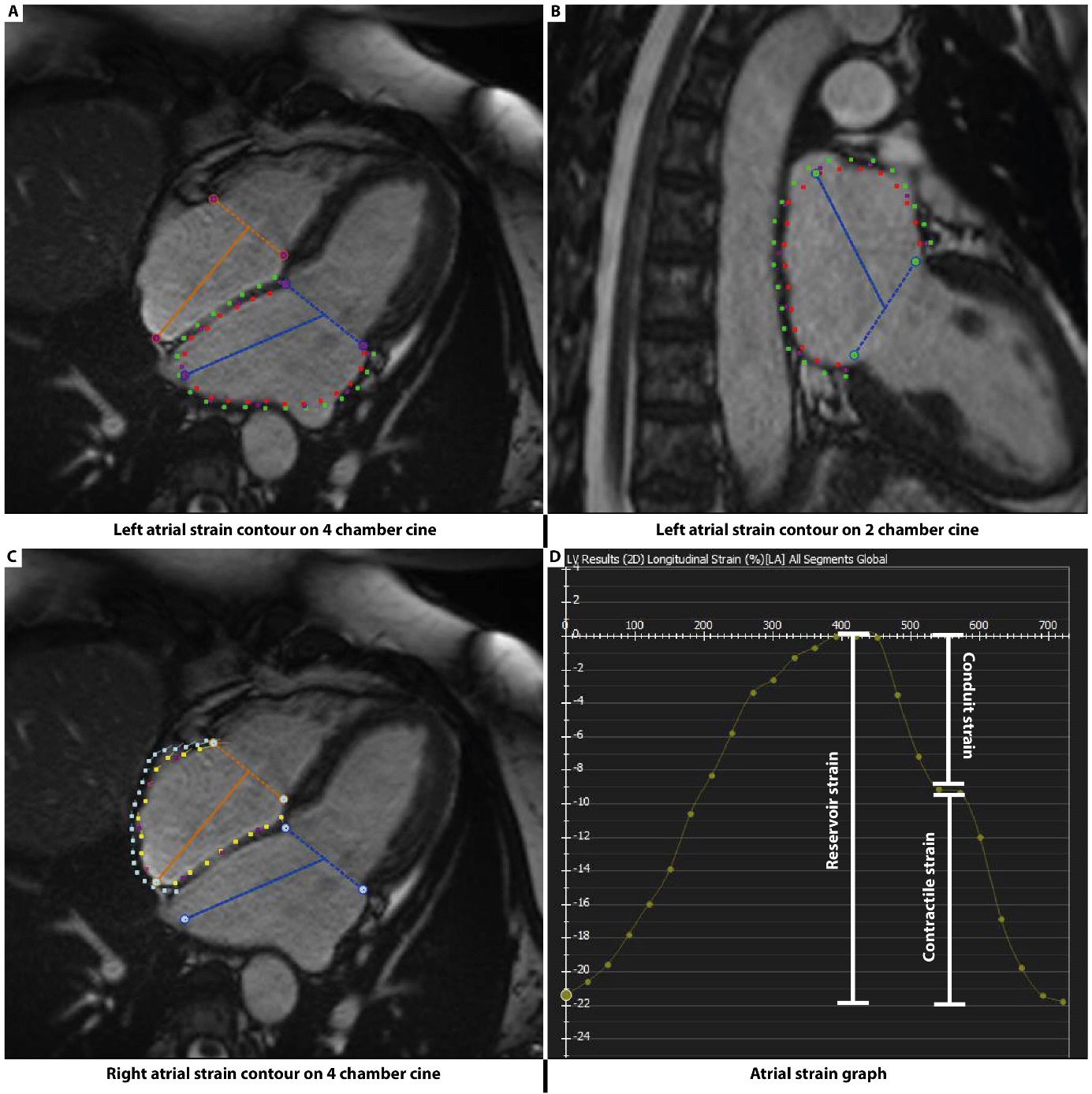
Figure S1:** Left and right atrial strain assessment

**A)** Four-chamber view showing the RA endocardial and epicardial contours. **B)** Four-chamber view showing the LA endocardial and epicardial contours. **C)** Two-chamber view showing the LA endocardial and epicardial contours. **D)** Atrial strain curve illustrating the reservoir phase, conduit phase and contractile phase.


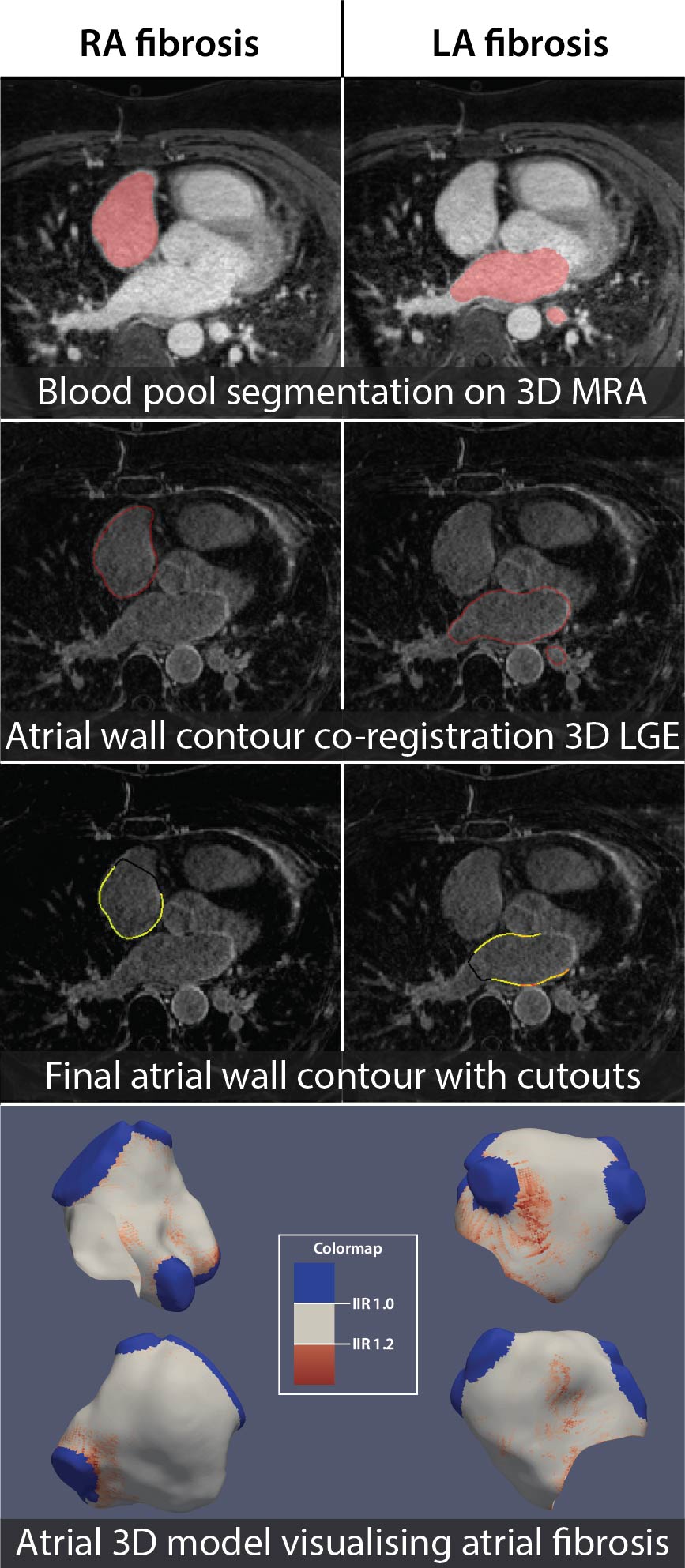
**Figure S2:** Left and right atrial fibrosis quantification

Pannel demonstrating the stepwise segmentation process for quantification of LA and RA fibrosis. *IIR: image intensity ratio, LGE: late gadolinium enhancement, MRA: Magnetic resonance angiography.*
